# Atlas of Clinical Medicine

**Published:** 1891-12

**Authors:** 


*Atlas of Clinical Medicine.* By BYROM BRAMWELL, M.D., F.R.C.P.E., F.R.S.E. Volume I. Part II. Edinburgh: T. AND A. CONSTABLE. 1891.

The first part of this work was so good that we hardly expected that it would be surpassed by the succeeding ones, but in our opinion this second part excels the first. In it Addison's disease, diffuse melanotic sarcoma, and Hodgkin's disease are treated of. The nature and origin of these remarkable diseases are still to a large extent unknown, and they are thereby invested with constant interest. Dr. Bramwell's clear and full account of them embraces the latest researches, and is most comprehensive, discussing them in all their bearings. He states lucidly the various theories that are held as to their pathogeny, and criticises the arguments on which these theories are founded. In his account of Addison's disease he has incorporated, as their importance demanded, the recent researches of MM. Alezais and Arnaud on the morbid changes in the pericapsular sympathetic ganglia of the suprarenals. Altogether this is the best account of Addison's disease known to us. Besides the plates directly illustrating the letter-press of this part, there is a crayon drawing of a case of mania, a coloured representation of xeroderma pigmentosum, and a plate of a remarkable case of molluscum fibrosum. All the illustrations are exceptionally good, and, apart from their great value and interest as accurate representations of disease, are of artistic excellence.

---

Our attention has been called to the fact that an extra edition, wholly in English, has been printed of the *Bibliotheca Medico-Chirurgica* (Vandenhoeck & Ruprecht, Göttingen), which we reviewed in September. Messrs. Luzac & Co., of London are prepared to receive subscriptions for this, which, under the title of *International Medical Bibliography*, is issued at a low price, in the hope that a sufficient number of English-speaking subscribers will be found to ensure its continuance.

